# Intergenerational nutrition benefits of India’s national school feeding program: Reality or a bridge too far?

**DOI:** 10.1038/s41467-022-33338-1

**Published:** 2022-10-27

**Authors:** Harshpal Singh Sachdev, Clive Osmond, Anura V. Kurpad, Tinku Thomas

**Affiliations:** 1 Pediatrics and Clinical Epidemiology, Sitaram Bhartia Institute of Science and Research, New Delhi, India; 2 Emeritus Professor of Biostatistics, MRC Lifecourse Epidemiology Unit, University of Southampton, Southampton, UK; 3 Department of Physiology, St John’s Medical College, Bengaluru, India; 4 Department of Biostatistics, St John’s Medical College, Bengaluru, India

**Keywords:** Developing world, Public health, Epidemiology, Nutrition

**arising from** S. Chakrabarti et al. *Nature Communications* 10.1038/s41467-021-24433-w (2021)

Chakrabarti et al.^1^ need to be complimented for their thorough analyses, to the extent that is possible with national datasets, to determine whether India’s school feeding program (the mid-day meal, MDM) has led to intergenerational nutrition benefits. However, given the nature of the analysed data and modelling, it is possible for the conclusions to be misleading or overreaching. The overreach is potentially adverse, as we show in the last paragraph of this matter arising from the publication.

The problem is that the same level of MDM exposure is attributed to all mothers within a state SES and birth cohort cell; its variability therein is not considered. The main result reported in the Abstract states: “height-for-age z-score (HAZ) among children born to mothers with full MDM exposure was greater (0.40 SD) than that in children born to non-exposed mothers”. This estimate and its precision should be considered with caution because it is based on 0–100% increase in MDM exposure, whereas the “real” benefit from the observed average increase in exposure was about one-third of that. Chakrabarti et al.^1^ also report the “real” population-wide effects of considering a 32% expansion in MDM, but highlighting the 0.4 SD increase as the main result seems an overreach.

In a local context, the proportion of primary school children covered with Mid-Day Meal (MDM) program (*exposure*) could be a marker of favourable socio-demographic or developmental characteristics (for example, maternal education or empowerment, household wealth, income, water supply and sanitation facilities, caste, religion, etc.) that are associated with greater height-for-age Z (HAZ) scores (*outcome*). The Controlled Interrupted Time Series design cannot eliminate this potential for allocation bias. Maternal height and education were not included in the final model since they were taken as mediating variables. However, a mediation analysis could have been attempted to draw estimates of the mediated effect of MDM through height and education (and other mediating variables), thus being able to analyse the intergenerational pathway in a better manner, such that their true variability and contribution to attained offspring height could be quantified. Perhaps, stratified analysis by maternal height would be helpful, notwithstanding the challenges of accounting for confounding.

Evidence supports the plausibility of an alternative hypothesis. First, in comparison to the control group, the intervention (MDM) group due to allocation bias had participants with favourable socio-demographic characteristics, which are associated with higher child HAZ, resulting in larger mothers who bore taller children. In this study too, the child HAZ was lower (overlapping 95% CI) among control mothers prior to the expansion of MDM (Fig. 5b^1^). Further, a unique comparison of the heights of children with that of their parents at corresponding ages from the New Delhi Birth Cohort, endorses this contention^2^. The parents were born between 1969–1972 and all had reached the age of 10 years by 1982, much before the MDM scheme was introduced nationally. The parent’s (father or mother) height at corresponding age was the strongest independent predictor (beta-coefficient: 0.53; 95% CI 0.43, 0.66) of *attained* height of the child^3^. Second, the hypothesised cycle above may be amplified by the intergenerational continuation of these favourable socio-demographic characteristics. “The India Human Development Surveys show that a household generally only moves up by one or two Socio-Economic-Status (SES) deciles over 7 years, if they move at all” (relatively static), and that “MDM coverage within states does not fluctuate greatly with small increments of SES classes”^1^.

The authors have attempted to control for several confounders and perform robustness checks. However, with more granular adjustments (birth year and state-specific SES fixed effects) or substituting the relatively time-invariant caste and religion for SES (Supplementary Fig. 4^1^), MDM was not significantly (*P* > 0.05) associated with child HAZ. While not belabouring the *P* value, it is important to note that confidence in the observed estimates, and thereby their translation, is attenuated. Further robustness checks would have been useful, including a correction for multiple testing and consideration of fathers along with mothers. It is also difficult to contemplate that a few years of exposure to a single intervention in an individual mother during her childhood, that is, a comparison of those who did or did not receive MDM (0% vs 100%), can lead to an average difference of 0.4Z in her child’s height.

An additional critique of this paper’s interpretation^1^ comes from examining the change in spatial distribution of the state under-five stunting prevalence, between NFHS-3 and NFHS-4 (Fig. 1). The spatial distribution of reduction in stunting is comparable across states, except for Chhattisgarh and Arunachal Pradesh. While Chhattisgarh was part of the MDM intervention group, Arunachal Pradesh was not: yet both showed a reduction in the prevalence of stunting between the two surveys. Indeed, stunting actually increased in Jharkhand, which did have the MDM introduced as an intervention. Thus, the modelled MDM intervention that was expected to disrupt the trend in stunting prevalence is not observed. Aggregate data-based outcome interpretation of state-specific changes in nutritional intake attributed to MDM, should therefore be made with abundant caution.

**Fig. 1:**
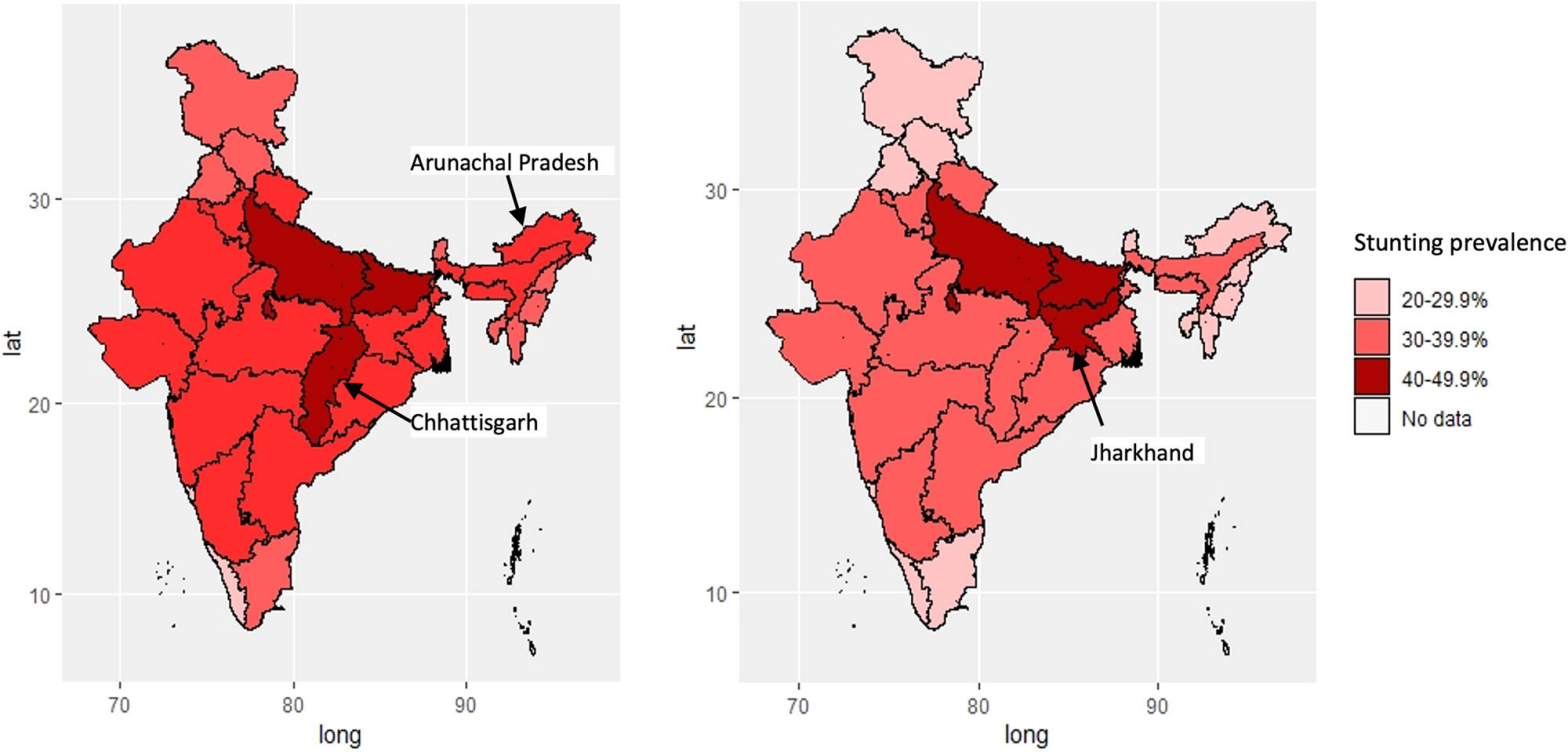
State-based stunting prevalence in <5-year-old children in two successive National Family Health Surveys: NFHS-3 (2005; left panel) and NFHS-4 (2015; right panel). The spatial distribution of reduction in stunting is comparable across states, except for Chhattisgarh and Arunachal Pradesh. While Chhattisgarh was a part of the MDM intervention group, Arunachal Pradesh was not: yet both showed a reduction in the prevalence of stunting between the two surveys. Indeed, stunting actually increased in Jharkhand, which did have MDM introduced as an intervention.

Finally, there is a real need for caution in interpretation. India is witnessing a rapid nutrition transition in tandem with an improvement in socio-demographic and economic indicators. Extreme caution is required in extending the school feeding programs beyond primary school, as hinted by the authors^1^. Recent data from the quality-controlled Comprehensive National Nutrition Survey from India show that among 5-19-years-old children and adolescents, “metabolic obesity” (dysglycemia or dyslipidemia) was present in 56% of participants^4^. Importantly, the prevalence was similar in anthropometrically undernourished (54% of thin and 59% of stunted) participants, and among the poor and rural inhabitants^4^. Ironically, in direct opposition to the objective of the school feeding program, the recommended core interventions^5^ for these metabolic perturbations, such as dietary restrictions and active lifestyle, are directed towards inducing a negative energy balance. This concern is underlined in the recent Indian nutrient recommendations^6^, where adult energy requirements have been revised downwards, in part based on low physical activity, and similarly, lower energy requirements have been suggested for sedentary children, since low physical activity levels were documented in 8–9-year-old Indian children^6,7^. Overenthusiastic feeding programmes, especially with low-quality cereal-dominated diets that are not unusual at national scale^8^, have the potential to fuel the ongoing epidemic of Non-Communicable Diseases and could result in disproportionate allocation for feeding subsidies in preference to other facets of development.

## Data Availability

All data used for the maps in figure are from the state level estimates of stunting in National Family Survey-Rounds 3 and 4 which are available at http://rchiips.org/nfhs/.
